# A Secrecy Transmission Protocol with Energy Harvesting for Federated Learning

**DOI:** 10.3390/s22155506

**Published:** 2022-07-23

**Authors:** Ping Xie, Fan Li, Ilsun You, Ling Xing, Honghai Wu, Huahong Ma

**Affiliations:** 1 School of Information Engineering, Henan University of Science and Technology, Luoyang 471023, China; xieping@haust.edu.cn (P.X.); lifan_12398@163.com (F.L.); xingling_my@163.com (L.X.); honghai2018@haust.edu.cn (H.W.); mhh@haust.edu.cn (H.M.); 2 Department of Financial Information Security, Kookmin University, Seoul 02707, Korea

**Keywords:** energy harvesting, federated learning, intercept probability, outage probability, secrecy transmission protocol

## Abstract

In federated learning (FL), model parameters of deep learning are communicated between clients and the central server. To better train deep learning models, the spectrum resource and transmission security need to be guaranteed. Toward this end, we propose a secrecy transmission protocol based on energy harvesting and jammer selection for FL, in which the secondary transmitters can harvest energy from the primary source. Specifically, a secondary transmitter ${\mathrm{ST}}_{i}$ is first selected, which can offer the best transmission performance for the secondary users to access the primary frequency spectrum. Then, another secondary transmitter ${\mathrm{ST}}_{n}$, which has the best channel for eavesdropping, is also chosen as a friendly jammer to provide secrecy service. Furthermore, we use outage probability (OP) and intercept probability (IP) as metrics to evaluate performance. Meanwhile, we also derive closed-form expressions of OP and IP of primary users and OP of secondary users for the proposed protocol, respectively. We also conduct a theoretical analysis of the optimal secondary transmission selection (OSTS) protocol. Finally, the performance of the proposed protocol is validated through numerical experiments. The results show that the secrecy performance of the proposed protocol is better than the OSTS and OCJS, respectively.

## 1. Introduction

In modern artificial intelligence, federated learning (FL) [1] is one of the most dominant collaborative training paradigms. Compared to traditional and centralized training methods, FL can mitigate the privacy leakage risk of data since the model parameters of clients are only transmitted to a central server in the training process. Most of the modern information and communication technologies [2] can satisfy the transmission of model parameters in the fifth generation (5G) networks [3]. Nevertheless, spectrum resource is essential for the transmission of model parameters in FL. Meanwhile, most of the spectrum resources are assigned by the government. Therefore, the spectrum resource is scarce for transmitting large amounts of information. For this reason, cognitive radio (CR) [4] is a promising technique to raise spectrum efficiency [5]. By integrating the advantages of Internet of Things (IoT) and CR, Cognitive Internet of Things (CIoT) becomes a prevalent network pattern. In CIoT, secondary users (SUs) can transmit information opportunistically without affecting legitimate users [6]. Moreover, resource utilization can be improved through intelligent cooperation [7].

However, active transmissions between clients and the central server in the framework of FL are vulnerable to eavesdropping by illegal users due to the essential nature of broadcast communication and dynamic spectrum access in CIoT. Thereby, how to ensure the transmission security and resist malicious intrusion [8] is a crucial problem in FL. To mitigate this problem, the physical-layer security (PLS) technology is an important protection mechanism [9,10,11,12]. In this direction, Csiszar and Korner [13] improved the security performance and broadcast private messages by leveraging the randomization of stochastic encoding. Tang et al. [14] used a helping interferer to improve the security of transmission. Specifically, the achieving secrecy rate can be also obtained even when the conditions of the destination channel are worse than the wiretap channel. Meanwhile, the perfect secrecy capacity of the multiple-input multiple-output (MIMO) channel was analyzed in [15]. Furthermore, the security performance of a multi-antenna system was also improved in [16,17]. In addition, some studies [18,19,20] have improved PLS by transmitting artificial noise (AN) to hinder eavesdroppers. Moreover, the confidentiality of legitimate users was also improved by selecting an appropriate friendly jammer to transmit AN [21]. The aforementioned efforts were made for the simple traditional system model; however, how to guarantee the secrecy performance of primary users (PUs) with strict requirements of Quality-of-Service (QoS) remains a key issue.

Meanwhile, the above-mentioned works focus on the secrecy performance of wireless communication without considering energy efficiency, which is a key problem in CIoT [22,23,24,25]. To enhance the energy efficiency, one of the most dominant methods is Energy Harvesting (EH), which harvests energy from the surroundings and prolongs the service life of wireless networks [26,27,28]. Furthermore, the performance of cognitive wireless networks with EH has been studied in recent years. For example, the secondary outage probability (OP) of EH cognitive radio systems was investigated in [29,30], where the opportunity relay selection (ORS) was used to select the best relay collaborative information transmission. In addition, some researchers are devoted to maximizing the throughput of EH cognitive radio networks, in which SUs collect energy from PUs. Specifically, Zheng et al. [31] considered three typical scenarios under the two cooperation modes of energy and joint mode to explore the factors affecting throughput. Furthermore, Liu et al. [32] analyzed the factors affecting the final decision threshold (*k*) and developed the optimal cooperative spectrum sensing (CSS) strategy according to the appropriate *k* value to maximize the throughput.

At present, EH combined with PLS has also attracted widespread attention [33,34,35]. Reference [33] adopted the relay protocol based on time switching, and EH technology is used to assist the relay and jammer to transmit secret and jamming signals. The security outage probability of the system is studied through two relay and jammer selection schemes. Reference [34] investigated the PLS of energy-harvesting cognitive radio networks and compared the security–reliability tradeoff (SRT) performance of the channel-aware user scheduling (CaUS) and energy-aware user scheduling (EaUS) methods. Reference [35] further analyzed the SRT of an energy-harvesting cooperative cognitive radio system and proposed two relay selection schemes to improve the security of cognitive users. Table 1 is a summary of some related works. The above works were committed to using EH to enhance the power of SUs or relays so as to assist SUs in transmitting data. Nonetheless, how to use energy harvesting to raise the confidentiality performance of PUs remains an open problem in FL over CIoT.

**Figure 1:**
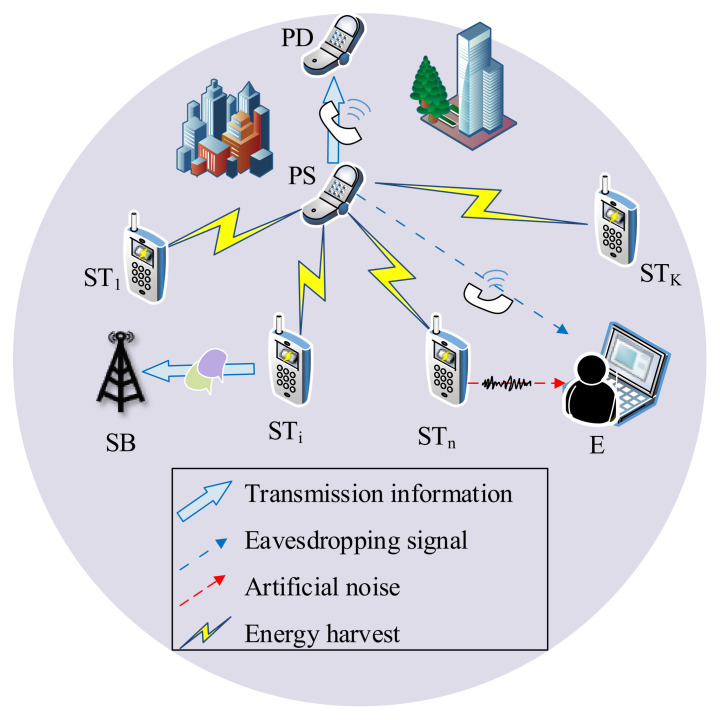
System scenario.

To improve the confidentiality performance of PUs, we integrate the advantages of the PLS technology and EH method into CIoT, which consists of multiple secondary transmitters (STs). The system scenario is shown in Figure 1. Meanwhile, we propose an ST transmission protocol by using cooperative transmission and friendly jamming. In the proposed protocol, STs obtain energy from the primary source (PS) in the first stage of the transmission slot and then transmit the signal in the second stage of the transmission slot to improve energy efficiency and spectrum utilization. In other words, a secondary transmitter (ST), which meets the interference threshold and can offer the best information transmission for SUs, is first chosen to share the PUs’ spectrum. Another ST, which meets the interference threshold and offers the optimal security performance for PUs, is then chosen to transmit AN. To invigorate ST as the friendly jammer, the interference threshold for SUs is relaxed by the PUs.

The mainly contributions of this paper are summarized as follows:

- We propose a secrecy transmission protocol based on Energy Harvesting (EH) and jammer selection to improve the PLS of PUs for FL, where the AN is transmitted by a cooperative jammer to obstruct eavesdroppers. Moreover, the influence of the basic power of the secondary transmitter on EH and the primary users is considered. In addition, the secondary outage performance is enhanced due to cooperation compensation and multi-user diversity gain.
- A dual secondary transmitter selection scheme is proposed to determine the secondary signal transmitter and friendly jammer. The ST that can offer the smallest OP is selected to transmit model parameters. Thus, the secondary transmission performance is enhanced by the ST selection. Another ST that can provide the smallest intercept probability (IP) is selected to transmit AN. Therefore, the primary security performance is enhanced by the friendly jammer selection.
- To compare the proposed protocol with optimal secondary transmission selection (OSTS) protocol, we derived the closed-form expressions of OP and IP of PUs and OP of SUs over Rayleigh fading channel for the above two protocols, respectively.
- The simulation results show that our protocol achieves better security performance than the OSTS and Optimal Cooperative Jammer Selection (OCJS) methods. Moreover, the secondary outage probabilities of the proposed scheme are lower than the OSTS and OCJS in high primary SNR, respectively. Furthermore, we improve the confidentiality of PUs and explore the influence of different parameters on the security performance.

The remainder of the paper is summarized as follows. An Energy-Harvesting Cognitive underlay system model and OSTS model is presented in Section 2. Section 3 presents OP and IP analysis for the cooperation transmission protocol. The OP and IP are analyzed for the OSTS model in Section 4. The numerical results of the performance comparison between the two methods are shown in Section 5. Finally, Section 6 contains the summary.

Notations: $|h_{P}{|}^{2}$, $|h_{\mathrm{PE}}{|}^{2}$, $|h_{{\mathrm{PS}}_{n}}{|}^{2}$, $|h_{S_{n}E}{|}^{2}$, $|h_{S_{i}E}{|}^{2}$, $|h_{{\mathrm{PS}}_{i}}{|}^{2}$, $|h_{S_{i}D}{|}^{2}$, $|h_{S_{i}}{|}^{2}$, and $|h_{\mathrm{PB}}{|}^{2}$ mean the channel coefficients from PS→PD, PS→E, PS→ST ${}_{n}$, ST ${}_{n}$→E, ST ${}_{i}$→E, PS→ST ${}_{i}$, ST ${}_{i}$→PD, ST ${}_{i}$ →SB, and PS→SB, respectively. All channels in this paper are considered to experience quasi-static Rayleigh fading, and the channel gain coefficient $|h_{\nu }{|}^{2}$ is regarded as an independently exponentially distributed random variable with a mean of $\sigma_{v}^{2}$. Namely, the Probability Density Function (PDF) of $|h_{\nu }{|}^{2}$ is expressed as follows:

(1) $$\begin{array}{c} f_{|h_{\nu }{|}^{2}}\left(x\right)=\frac{1}{\sigma_{\nu }^{2}}\exp\left(-\frac{x}{\sigma_{\nu }^{2}}\right), \end{array}$$

where $\nu \in \{\mathrm{PE},{\mathrm{PS}}_{n},S_{n}E,S_{i}E,{\mathrm{PS}}_{i},S_{i}D,S_{i},\mathrm{PB}\}$. $R_{P}$ and $R_{S}$ mean the minimum data rates of PUs and SUs, respectively. $P_{P}$ and $P_{S_{i}}$ represent the transmit powers of PS and ST ${}_{i}$, respectively. We assume that the received noises of all receivers are zero-mean Additive White Gaussian Noises (AWGNs) with a variance of $N_{0}$. Pr{X} and E[X] mean the probability and expected value of an event *X*.

## 2. System Model Descriptions

### 2.1. The Energy-Harvesting Cognitive Underlay System Model

**Figure 2:**
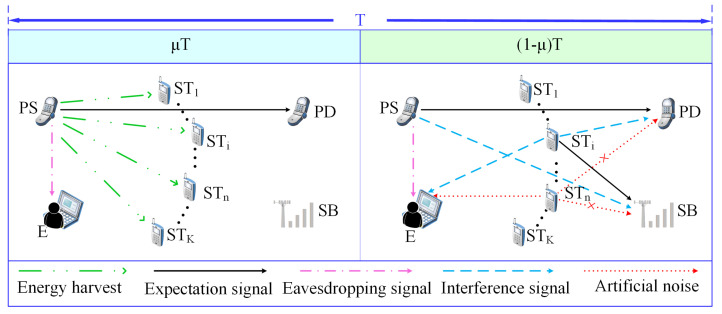
An energy-harvesting cognitive underlay system model. ( μT: the segment slot for energy harvest and only primary signal transmission. (1−μ)T: the segment slot for primary and secondary signals transmission. )

We consider an Energy-Harvesting Cognitive underlay system model, which is comprised of a primary pair (PS-PD), an eavesdropper (E), a secondary base station (SB) and *K* secondary transmitters (${\mathrm{ST}}_{i}$, where $i\in O=\left\{1,2,\ldots ,K\right\}$). The model is shown in Figure 2. In this model, secondary users can simultaneously access the licensed band with the primary system as along as the QoS of primary user is able to guarantee. Because of the battery-limited nature of ${\mathrm{ST}}_{i}$, the EH technology is utilized to extend the network lifetime. The eavesdropper is very interested in the primary information. Thus, it tries to overhear and tap the active transmissions of the PS all the time. Moreover, the model can be applied to device-to-device (D2D) communication scenarios, and D2D users equipped with energy harvesters can play as the friendly jammers.

In the proposed protocol, one secondary transmitter denoted by ${\mathrm{ST}}_{i}$, which can provide the best secondary outage performance, is selected to deliver secondary signals. Another secondary transmitter denoted by ${\mathrm{ST}}_{n}$, which can provided the best primary intercept performance, is selected to transmit AN, where i,n∈O and i≠n. The AN is produced by pseudo-random sequences known to PD and SB but unknown to E. Thus, AN makes no difference to PD and SB but causes serious influence to E. The signal transmission power at ${\mathrm{ST}}_{i}$ is determined by the combination of the harvesting energy, initial energy, and interference threshold. For the selfishness of ST, however, the signal transmission power at ${\mathrm{ST}}_{n}$ is just determined by the harvesting energy. The detailed transmission mechanism and secondary transmitter selection scheme are introduced in the next subsection.

### 2.2. Information Transmission

In this paper, the message transmission mechanism of the primary system is the same as that in traditional underlay cognitive networks, i.e., the primary data are continuously transmitted over the entire time slot. Moreover, the time slot receiver protocol for EH and information transmission at ${\mathrm{ST}}_{i}$ is employed, which is also used in [36]. Specifically, the total communication time slot consists of two segments: ${\mathrm{ST}}_{i}$ collects energy from PS in the front segment denoted as μT and transmits secondary information or artificial noise in the back segment denoted as (1−μ)T, where 0≤μ≤1 represents the slot split ratio and *T* represents the total length of each time slot. According to [37], the gathering energy at ${\mathrm{ST}}_{i}$ in the front segment slot can be presented as follows:

(2) $$\begin{array}{c} E_{ST_{i}}=\eta \mu TP_{P}{\left|h_{PS_{i}}\right|}^{2}, \end{array}$$

where 0≤η≤1 means the energy transfer efficiency. Meanwhile, a collection of working secondary transmitters that meet the interference threshold is expressed as *Q* [38]. When Q=∅, only PS transmits the signals; the secondary transmission is interrupted. Thus, the received signals at PD and E are expressed as in (3) and (4), respectively. The instantaneous capacities of the PS→PD link and the PS→E link are expressed as in (5) and (6), respectively.

(3) $$\begin{array}{c} r_{P1}\left(t\right)=\sqrt{P_{P}}h_{P}x_{P}\left(t\right)+n_{P}\left(t\right), \end{array}$$

(4) $$\begin{array}{c} r_{E1}\left(t\right)=\sqrt{P_{P}}h_{\mathrm{PE}}x_{P}\left(t\right)+n_{E}\left(t\right), \end{array}$$

(5) $$\begin{array}{c} C_{P1}=\mu T\log_{2}\left(1+\frac{P_{P}{\left|h_{P}\right|}^{2}}{N_{0}}\right), \end{array}$$

(6) $$\begin{array}{c} C_{E1}=\mu T\log_{2}\left(1+\frac{P_{P}{\left|h_{\mathrm{PE}}\right|}^{2}}{N_{0}}\right). \end{array}$$

It assumes that the initial energy owned by ${\mathrm{ST}}_{i}$ can be expressed as $E_{0}=P_{0}T$, where $P_{0}$ is the basic transmission power. On the one hand, the transmitted power at ${\mathrm{ST}}_{i}$ in the (1−μ)T segment slot depends on the combination of the harvesting energy and initial energy. On the other hand, the interference to PD caused by ${\mathrm{ST}}_{i}$ must be lower than the maximum tolerable interference level that is denoted by *I* in the underlay cognitive model. Thus, the transmitted power at ${\mathrm{ST}}_{i}$ in the (1−μ)T segment slot can be expressed as

(7) $$\begin{array}{c} P_{S_{i}}=\min\left(\frac{I}{{\left|h_{S_{i}D}\right|}^{2}},\frac{\eta \mu P_{P}{\left|h_{PS_{i}}\right|}^{2}+P_{0}}{1-\mu }\right). \end{array}$$

When Q≠∅, PS transmits the primary signals. Meanwhile, in the back segment slot, secondary signals are transmitted by ${\mathrm{ST}}_{i}$ to SB and an artificial noise is delivered by ${\mathrm{ST}}_{n}$. Therefore, the received signals at PD, SB, and E are expressed as

(8) $$\begin{array}{c} r_{P2}\left(t\right)=\sqrt{P_{P}}h_{P}x_{P}\left(t\right)+\sqrt{P_{S_{i}}}h_{S_{i}D}x_{S}\left(t\right)+n_{P}\left(t\right), \end{array}$$

(9) $$\begin{array}{c} r_{S_{i}}\left(t\right)=\sqrt{P_{S_{i}}}h_{S_{i}}x_{S}\left(t\right)+\sqrt{P_{P}}h_{\mathrm{PB}}x_{P}\left(t\right)+n_{S_{i}}\left(t\right), \end{array}$$

(10) $$\begin{array}{cc} r_{E2}\left(t\right) & =\sqrt{P_{P}}h_{\mathrm{PE}}x_{P}\left(t\right)+\sqrt{P_{S_{n}}}h_{S_{n}E}x_{n}\left(t\right)+\sqrt{P_{S_{i}}}h_{S_{i}E}x_{S}\left(t\right)+n_{E}\left(t\right), \end{array}$$

where $P_{S_{n}}$ is the transmitted power at ${\mathrm{ST}}_{n}$. Because the energy at ${\mathrm{ST}}_{n}$ is limited and the AN makes no difference to PD, $P_{S_{n}}$ can be set to $\eta \mu P_{P}{\left|h_{{\mathrm{PS}}_{n}}\right|}^{2}/\left(1-\mu \right)$. $x_{n}\left(t\right)$, $x_{P}\left(t\right)$, and $x_{S}\left(t\right)$ indicate the AN, the PUs’ message symbol, and the SUs’ message symbol, respectively. Moreover, $n_{P}\left(t\right)$, $n_{S}\left(t\right)$, and $n_{E}\left(t\right)$ indicate noises at PD, SB, and E, respectively. Moreover, $E[{|x_{\alpha }\left(t\right)|}^{2}]=1$, where α∈{P,S,n}. According to the above conditions, the instantaneous capacities of the PS→PD link, the ${\mathrm{ST}}_{i}$→SB link, and the PS→E link transmission can be obtained by (11)–(13), respectively.

(11) $$C_{P2}^{}=\left(1-\mu \right)T\log_{2}\left(1+\frac{P_{P}{\left|h_{P}\right|}^{2}}{P_{S_{i}}{\left|h_{S_{i}D}\right|}^{2}+N_{0}}\right),$$

(12) $$C_{S_{i}}^{}=\left(1-\mu \right)T\log_{2}\left(1+\frac{P_{S}{\left|h_{S_{i}}\right|}^{2}}{P_{P}{\left|h_{\mathrm{PB}}\right|}^{2}+N_{0}}\right),$$

(13) $$C_{E2}^{}=\left(1-\mu \right)T\log_{2}\left(1+\frac{P_{P}{\left|h_{\mathrm{PE}}\right|}^{2}}{P_{S_{n}}{\left|h_{S_{n}E}\right|}^{2}+P_{S_{i}}{\left|h_{S_{i}E}\right|}^{2}+N_{0}}\right).$$

During the back segment slot, the optimal secondary signal transmitter ${\mathrm{ST}}_{i^{*}}$ and the optimal friendly jammer ${\mathrm{ST}}_{n^{*}}$ are selected due to the multi-user scheduling scheme. For optimal secondary reliable transmission performance, the optimal secondary signal transmitter ${\mathrm{ST}}_{i^{*}}$ can be selected via the ${\mathrm{ST}}_{i}$→SB link, i.e.,

(14) $$i^{*}=\arg\max_{i^{*}\in O}\left({\left|h_{S_{i}}\right|}^{2}\right).$$

Because the dual secondary transmitter selection is used, the optimal secondary signal transmitter cannot be played as the optimal friendly jammer, then i,n∈O and i≠n. For optimal primary security performance, the optimal friendly jammer ${\mathrm{ST}}_{n^{*}}$ can be selected via the ${\mathrm{ST}}_{n}$→E link, i.e.,

(15) $$n^{*}=\arg\max_{n^{*}\in O,n^{*}\neq i^{*}}\left({\left|h_{S_{n}E}\right|}^{2}\right).$$

### 2.3. The Optimal Secondary Transmission Selection Model

For comparison, we take the OSTS cognitive underlay model in [38] as the benchmark and further consider the battery-limited condition. The OSTS model consists of a primary pair (PS-PD), an eavesdropper (E), a secondary base station (SB) and *K* secondary transmitters (${\mathrm{ST}}_{i}$, i=1,…,K). The protocol also utilizes the friendly jamming technology to transmit AN and secondary signals by selecting an ST. There, the interference threshold for SUs is relaxed. Specifically, to linearly combine the AN with the secondary signal, the transmission power of ${\mathrm{ST}}_{i}$ is divided into ξ and 1−ξ, where 0≤ξ≤1 means the power distribution factor. Then, the combined signal can be expressed as $\sqrt{1-\xi }x_{n}\left(t\right)+\sqrt{\xi }x_{S}\left(t\right)$. Furthermore, the energy-harvesting technology is not considered in the model, while security–reliability trade-off can be employed according to [34]. Since the interference caused by ${\mathrm{ST}}_{i}$ must be lower than a threshold settled by the primary system in cognitive underlay models, the transmitted power at ${\mathrm{ST}}_{i}$ is limited to $P_{0}$ for the battery-limited condition; then, the transmitted power at ${\mathrm{ST}}_{i}$ can be expressed as

(16) $$P_{S_{i}}^{OSTS}=\min\left(I/|h_{S_{i}D}{|}^{2},P_{0}\right).$$

When only PS transmits the signals, the STs do not work (namely, $Q^{OSTS}=\emptyset$). The signals at PD and E are like (3) and (4), respectively. The instantaneous capacities of the PS→PD link and the PS→E link transmission can be expressed as in (17) and (18), respectively.

(17) $$C_{P1}^{OSTS}=\log_{2}\left(1+\frac{P_{P}{\left|h_{P}\right|}^{2}}{N_{0}}\right),$$

(18) $$C_{E1}^{OSTS}=\log_{2}\left(1+\frac{P_{P}{\left|h_{\mathrm{PE}}\right|}^{2}}{N_{0}}\right).$$

When $Q^{OSTS}\neq \emptyset$, the secondary signal and primary signal coexist in the licensed spectrum in the OSTS model. The received signals at PD, SB, and E are expressed as (19)–(21), respectively.

(19) $$\begin{array}{c} r_{P2}^{OSTS}\left(t\right)=\sqrt{P_{P}}h_{P}x_{P}\left(t\right)+\sqrt{\xi P_{S_{i}}^{OSTS}}h_{S_{i}D}x_{S}\left(t\right)+n_{P}\left(t\right), \end{array}$$

(20) $$\begin{array}{c} r_{S_{{}_{i}}}^{OSTS}\left(t\right)=\sqrt{\xi P_{S_{i}}^{OSIS}}h_{S_{i}}x_{S}\left(t\right)+\sqrt{P_{P}}h_{\mathrm{PB}}x_{P}\left(t\right)+n_{S_{i}}\left(t\right), \end{array}$$

(21) $$\begin{array}{c} r_{E2}^{OSTS}\left(t\right)=\sqrt{P_{P}}h_{\mathrm{PE}}x_{P}\left(t\right)+\left[\sqrt{\left(1-\xi \right)P_{S_{i}}^{OSTS}}x_{n}\left(t\right)+\sqrt{\xi P_{S_{i}}^{OSTS}}x_{S}\left(t\right)\right]h_{S_{i}E}+n_{E}\left(t\right). \end{array}$$

Thus, the instantaneous capacities of the PS→PD link, the ${\mathrm{ST}}_{i}$→SB link, and the PS→E link transmission can be written as (22)–(24), respectively.

(22) $$C_{P2}^{OSTS}=\log_{2}\left[1+\frac{P_{P}{\left|h_{P}\right|}^{2}}{\xi P_{S_{i}}^{OSTS}{\left|h_{S_{i}D}\right|}^{2}+N_{0}}\right],$$

(23) $$C_{S}^{OSTS}=\log_{2}\left[1+\frac{\xi P_{S_{i}}^{OSTS}{\left|h_{S_{i}}\right|}^{2}}{P_{P}{\left|h_{\mathrm{PB}}\right|}^{2}+N_{0}}\right],$$

(24) $$C_{E}^{OSTS}=\log_{2}\left[1+\frac{P_{P}{\left|h_{\mathrm{PE}}\right|}^{2}}{P_{S_{i}}^{OSTS}{\left|h_{S_{i}E}\right|}^{2}+N_{0}}\right].$$

As is well known, multi-user diversity technology can effectively improve the performance of communication systems. Similar to [36], a security–reliability trade-off can be used to enhance the security performance of the OSTS model. Furthermore, the selection criteria for ${\mathrm{ST}}_{i^{*}}$, which may share PUs’ spectrum for transmitting secondary signals, can be shown as

(25) $$ST_{i*}=\arg\min_{i*\in O}\mathrm{Pr}\left\{C_{S}^{OSTS}<R_{S}\right\}=\arg\max_{i*\in O}\left({\left|h_{S_{i}E}\right|}^{2}\right).$$

## 3. The OP and IP Analysis for the Cooperation Transmission and Energy-Harvesting Protocol

As described in [39,40], OP and IP are two vital parameters to judge the reliability and secrecy of information transmission in communication. Therefore, we analyze these two parameters in detail.

### 3.1. The Primary OP Analysis

As described in [38], when Q≠∅, we denote $Q=Q_{l}$. In that case, both PS and ${\mathrm{ST}}_{i}$ transmit signals, where ${\mathrm{ST}}_{i}\in Q_{l}$ and $l=1,2,\ldots ,2^{K}-1$. The amount of elements in the collection $Q_{l}$ is *L*. $Q_{l}=\left\{ST_{i}|C_{P2}\geq R_{P},i\in \left\{1,\ldots ,K\right\}\right\}$ and $\bar{Q_{l}}=\left\{ST_{i}|C_{P2}<R_{P},i\in \left\{1,\ldots ,K\right\}\right\}$. $Q_{l}\cup \bar{Q_{l}}=\left\{ST_{1},ST_{2},\ldots ,ST_{K}\right\}$. $\Psi_{P}$ is defined as the transmission outage event of a primary system. We know that the event $\Psi_{P}$ is considered to happen when $C_{P2}<R_{P}$. The OP of PUs can be given by

(26) $$\begin{array}{c} P_{out}=\mathrm{Pr}\left\{Q=\emptyset \right\}\mathrm{Pr}\left\{\Psi_{P}|Q=\emptyset \right\}+\sum_{l=1}^{2^{K}-1}\mathrm{Pr}\left\{Q=Q_{l}\right\}\mathrm{Pr}\left\{\Psi_{P}|Q=Q_{l}\right\}. \end{array}$$

After that, $\mathrm{Pr}\left\{Q=\emptyset \right\}$ can be shown as

(27) $$\begin{array}{cc} \mathrm{Pr}\left\{Q=\emptyset \right\} & =\prod_{i=1}^{K}\mathrm{Pr}\left\{C_{P2}<R_{P}\right\}\\ \; & =\prod_{i=1}^{K}\mathrm{Pr}\left\{\left(1-\mu \right)T\log_{2}\left(1+\frac{P_{P}{\left|h_{P}\right|}^{2}}{P_{S_{i}}{\left|h_{S_{i}D}\right|}^{2}+N_{0}}\right)<R_{P}\right\}\\ \; & =\prod_{i=1}^{K}\mathrm{Pr}\left\{\frac{P_{P}{\left|h_{P}\right|}^{2}}{P_{S_{i}}{\left|h_{S_{i}D}\right|}^{2}+N_{0}}<2^{\frac{R_{P}}{\left(1-\mu \right)T}}-1\right\}, \end{array}$$

where $C_{P2}$ is given by (11). According to the results given by (A1)–(A3) in Appendix A, the final expression of $\mathrm{Pr}\left\{Q=\emptyset \right\}$ is

(28) $$\mathrm{Pr}\left\{Q=\emptyset \right\}=\prod_{i=1}^{K}\mathrm{Pr}\left\{\mathrm{Ch}1\times \mathrm{Ch}2\right\}$$

Moreover, $\mathrm{Pr}\left\{\Psi_{P}|Q=\emptyset \right\}$ and $\mathrm{Pr}\left\{Q=Q_{l}\right\}$ can be calculated by (29) and (30), respectively.

(29) PrΨP|Q=∅=PrμTlog21+PPhP2N0<RP=1−exp(−N02RPRPμTμT−1PPσP2),

(30) $$\begin{array}{cc} \mathrm{Pr}\left\{Q=Q_{l}\right\} & =\prod_{i\in \bar{Q_{l}}}\mathrm{Pr}\left\{C_{P2}<R_{P}\right\}\prod_{j\in Q_{l}}\mathrm{Pr}\left\{C_{P2}\geq R_{P}\right\}\\ \; & =\prod_{i\in \bar{Q_{l}}}\left(\mathrm{Ch}1\times \mathrm{Ch}2\right)\prod_{j\in Q_{l}}\left(1-\mathrm{Ch}1\times \mathrm{Ch}2\right). \end{array}$$

According to the definition of the collection $Q_{l}$, we can know that $\mathrm{Pr}\left\{\Psi_{P}|Q=Q_{l}\right\}=0$. Thus, the OP of PUs is derived by substituting (28)–(30) and substituting $\mathrm{Pr}\left\{\Psi_{P}|Q=Q_{l}\right\}=0$ into (26). Here, Ch 1 and Ch 2 are calculated by (A2) and (A3), respectively.

### 3.2. The Secondary OP Analysis Based on Optimal Selection Strategy

As described in [38], $\Psi_{S}$ is defined as the transmission outage event of a secondary system. The event $\Psi_{S}$ is indicated to happen when $C_{S_{i}}<R_{P}$. In addition, the event $\Psi_{S}$ will happen when all STs do not work, i.e., Q=∅ or the QoS of secondary users is not satisfied (Q≠∅). In the proposed protocol based on an optimal selection strategy, an optimal ${\mathrm{ST}}_{i}$ is selected to transmit the secondary information, which can provide the best secondary transmission performance. The secondary outage probability of the proposed protocol based on optimal selection strategy can be written as

(31) $$\begin{array}{c} S_{out}^{opt}=\mathrm{Pr}\left\{Q=\emptyset \right\}\mathrm{Pr}\left\{\Psi_{S}|Q=\emptyset \right\}+\sum_{l=1}^{2^{K}-1}\mathrm{Pr}\left\{Q=Q_{l}\right\}\mathrm{Pr}\left\{\Psi_{S}|Q=Q_{l}\right\}. \end{array}$$

Furthermore, $\mathrm{Pr}\left\{\Psi_{S}|Q=Q_{l}\right\}$ can be calculated as

(32) $$\begin{array}{cc} \; & \mathrm{Pr}\left\{\Psi_{S}|Q=Q_{l}\right\}\\ \; & =\mathrm{Pr}\left\{\left(1-\mu \right)T\log_{2}\left(1+\frac{P_{S_{i}}\max\left({\left|h_{S_{i}}\right|}^{2}\right)}{P_{P}{\left|h_{\mathrm{PB}}\right|}^{2}+N_{0}}\right)<R_{S}\right\}\\ \; & =\prod_{i=1}^{L}\mathrm{Pr}\left\{\frac{P_{S_{i}}{\left|h_{S_{i}}\right|}^{2}}{P_{P}{\left|h_{\mathrm{PB}}\right|}^{2}+N_{0}}<2^{\frac{R_{S}}{\left(1-\mu \right)T}}-1\right\}. \end{array}$$

According to the results given by (A4)–(A6) in Appendix A, the final expression of $\mathrm{Pr}\left\{\Psi_{S}|Q=Q_{l}\right\}$ is

(33) $$\mathrm{Pr}\left\{\Psi_{S}|Q=Q_{l}\right\}=\prod_{i=1}^{K}\mathrm{Pr}\left\{1-\mathrm{Ch}3\times \mathrm{Ch}4\right\}$$

We know that $\mathrm{Pr}\left\{\Psi_{S}|Q=\emptyset \right\}=1$. The OP of SUs in our protocol based on the optimal selection strategy for ${\mathrm{ST}}_{i^{*}}$ can be obtained by substituting (28)–(33), and $\mathrm{Pr}\left\{\Psi_{S}|Q=\emptyset \right\}=1$ into (31). Here, Ch 1–Ch 4 are calculated by (A2)–(A6), respectively.

### 3.3. The Primary IP Analysis Based on Optimal Selection Strategy

According to [38], $\Psi_{\mathrm{int}}$ denotes the transmission intercept event of PUs. Furthermore, $\Psi_{\mathrm{int}}$ is implied to occur when $R_{P}<C_{E2}$. When Q≠∅, the event $\Psi_{\mathrm{int}}$ may happen. Since i,n∈O and i≠n, the number of secondary transmitters selected at this condition is K−1. Let M=K−1, where $Q_{l}\cup \bar{Q_{l}}=\left\{ST_{1},ST_{2},\ldots ,ST_{M}\right\}$. Hence, the primary intercept probability of the proposed protocol based on the optimal selection strategy can be written as

(34) $$\begin{array}{cc} P_{\mathrm{int}}^{opt} & =\mathrm{Pr}\left\{Q=\emptyset \right\}\mathrm{Pr}\left\{\Psi_{\mathrm{int}}|Q=\emptyset \right\}+\sum_{l=1}^{2^{M}-1}\mathrm{Pr}\left\{Q=Q_{l}\right\}\mathrm{Pr}\left\{\Psi_{\mathrm{int}}|Q=Q_{l}\right\}. \end{array}$$

Next, $\mathrm{Pr}\left\{\Psi_{\mathrm{int}}|Q=\emptyset \right\}$ can be shown as

(35) PrΨint|Q=∅=PrCE1≥RP=PrμTlog21+PPhPE2N0≥RP=PrPPhPE2N0≥2RPμT−1=exp(−N02RPRPμTμT−1PPσPE2).

As described in (15), ${\mathrm{ST}}_{n^{*}}$, which has the best channel state conditions to E, is selected to transmit artificial noises to interfere with eavesdropping. Thus, $\mathrm{Pr}\left\{\Psi_{\mathrm{int}}|Q=Q_{l}\right\}$ can be derived as

(36) PrΨint|Q=Ql=PrCE2≥RP=PrPPhPE2PSn*hSn*E2+PSihSiE2+N0≥2RP1−μT−1=∏n=1LPrPPhPE2PSnhSnE2+PSihSiE2+N0≥ΔP=∏n=1LPrPSnhSnE2+PSihSiE2+N0PPhPE2≤1ΔP=∏n=1L1−PrminIhSiD2,ημPPhPSi2+P01−μhSiE2+ημPPhPSn2hSnE21−μ+N0=>PPhPE2ΔP=∏n=1L1−PrIhSiE2hSiD2+ημPPhPSn2hSnE21−μ+N0>PPhPE2ΔP︸Ch5=×PrημPPhPSi2+P0hSiE2+ημPPhPSn2hSnE21−μ+N0>PPhPE2ΔP︸Ch6.

To sum up, the IP of PUs based on the optimal selection strategy for ${\mathrm{ST}}_{n^{*}}$ can be obtained by substituting (28), (30), (35), and (36) into (34), where Ch 1, Ch 2, Ch 5, and Ch 6 are calculated by (A2), (A3), (A7), and (A8), respectively.

## 4. The OP and IP Analysis for the Battery-Limited OSTS Protocol

In the battery-limited OSTS protocol, the security–reliability trade-off is presented to enhance the primary security performance. Specifically, ${\mathrm{ST}}_{i^{*}}$, which has the best channel state conditions to SB, is selected for transmitting secondary data. Similar to the probability analysis of the proposed protocol, the OP of PUs and SUs, the IP of PUs are calculated by (37)–(39), respectively.

(37) PoutOSTS=PrQOSTS=∅PrΨP|QOSTS=∅=+∑l=12K−1PrQOSTS=QlPrΨP|QOSTS=Ql,

(38) SoutOSTS=PrQOSTS=∅PrΨS|QOSTS=∅=+∑l=12K−1PrQOSTS=QlPrΨS|QOSTS=Ql,

(39) PintOSTS=PrQOSTS=∅PrΨint|QOSTS=∅=+∑l=12K−1PrQOSTS=QlPrΨint|QOSTS=Ql,

where $\mathrm{Pr}\left\{\Psi_{S}|Q^{OSTS}=\emptyset \right\}=1$, $\mathrm{Pr}\left\{\Psi_{P}|Q^{OSTS}=Q_{l}\right\}=0$, and the rest of the probabilities in (37)–(39) are expressed by (40)–(46), respectively.

(40) $$\begin{array}{cc} \mathrm{Pr}\left\{\Psi_{P}|Q^{OSTS}=\emptyset \right\} & =\mathrm{Pr}\left\{\log_{2}\left(1+\frac{P_{P}{\left|h_{P}\right|}^{2}}{N_{0}}\right)<R_{P}\right\}=1-\exp(-\frac{N_{0}\rho_{P}}{P_{P}\sigma_{P}^{2}}), \end{array}$$

(41) $$\begin{array}{cc} \mathrm{Pr}\left\{Q^{OSTS}=\emptyset \right\} & =\prod_{i=1}^{K}\mathrm{Pr}\left\{C_{P2}^{OSTS}<R_{P}\right\}\\ \; & =\prod_{i=1}^{K}\mathrm{Pr}\left\{\log_{2}\left(1+\frac{P_{P}{\left|h_{P}\right|}^{2}}{\xi P_{S_{i}}^{OSTS}{\left|h_{S_{i}D}\right|}^{2}+N_{0}}\right)<R_{P}\right\}\\ \; & =\prod_{i=1}^{K}\mathrm{Pr}\left\{\frac{P_{P}{\left|h_{P}\right|}^{2}}{\xi P_{S_{i}}^{OSTS}{\left|h_{S_{i}D}\right|}^{2}+N_{0}}<2^{R_{P}}-1\right\}, \end{array}$$

where $\rho_{P}=2^{R_{P}}-1$ and $C_{P2}^{OSTS}$ are given by (22). According to the results given by (A9)–(A11) in Appendix A, the final expression of $\mathrm{Pr}\left\{Q^{OSTS}=\emptyset \right\}$ is

(42) $$\mathrm{Pr}\left\{Q^{OSTS}=\emptyset \right\}=\prod_{i=1}^{K}\mathrm{Pr}\left\{\mathrm{Ch}7\times \mathrm{Ch}8\right\}$$

According to (41) and (42), we have

(43) $$\begin{array}{cc} \mathrm{Pr}\left\{Q^{OSTS}=Q_{l}\right\} & =\prod_{i\in \bar{Q_{l}}}\mathrm{Pr}\left\{C_{P2}^{OSTS}<R_{P}\right\}\prod_{j\in Q_{l}}\mathrm{Pr}\left\{C_{P2}^{OSTS}\geq R_{P}\right\}\\ \; & =\prod_{i\in \bar{Q_{l}}}\left(\mathrm{Ch}7\times \mathrm{Ch}8\right)\prod_{j\in Q_{l}}\left(1-\mathrm{Ch}7\times \mathrm{Ch}8\right). \end{array}$$

In addition, $\mathrm{Pr}\left\{\Psi_{S}|Q^{OSTS}=Q_{l}\right\}$ can be written as follows:

(44) PrΨS|QOSTS=Ql=argmini*∈OPrCSOSTS<RS=PrξPSiOSTSmaxi*∈OhSiE2PPhPB2+N0<2RS−1=∏i=1LPrξPSiOSTShSiE2PPhPB2+N0<2RS−1=∏i=1L1−PrminIIhSiD2hSiD2,P0·ξhSi2>ρSPPhPB2+N0=∏i=1L1−PrξIhSi2ξIhSi2hSiD2hSiD2PPhPB2+N0>ρS︸Ch9·PrξP0hSi2PPhPB2+N0>ρS︸Ch10,

where $\rho_{S}=2^{R_{S}}-1$. Considering Y1=hSi2hSi2hSiD2hSiD2 and using the PDF of $Y_{1}$, which is calculated in Appendix B, Ch9 and Ch10 can be derived as (A12) and (A13), respectively.

Furthermore, $\mathrm{Pr}\left\{\Psi_{\mathrm{int}}|Q^{OSTS}=\emptyset \right\}$ can be derived as

(45) $$\mathrm{Pr}\left\{\Psi_{\mathrm{int}}|Q^{OSTS}=\emptyset \right\}=\mathrm{Pr}\left\{X_{6}\geq \frac{N_{0}\left(2^{R_{P}}-1\right)}{P_{P}}\right\}=e^{-\frac{N_{0}\rho_{P}}{P_{P}\sigma_{\mathrm{PE}}^{2}}}.$$

Following (25), the $\mathrm{Pr}\left\{\Psi_{\mathrm{int}}|Q^{OSTS}=Q_{l}\right\}$ can be written as (46), whileCh11 and Ch12 can be derived as (A14) and (A15), respectively.

(46) PrΨint|QOSTS=Ql=PrCEOSTS≥RP=PrPPhPE2PSiOSTShSiE2+N0≥2RP−1=1−PrPPhPE2<ρPPSiOSTShSiE2+N0=1−PrPPhPE2<ρPminIIhSiD2hSiD2,P0·hSiE2+N0=1−PrPPhPE2<ρPIhSiE2IhSiE2hSiD2hSiD2+N0︸Ch11=×PrPPhPE2<ρPP0hSiE2+N0︸Ch12.

## 5. Numerical Results

This section gives the simulation results of the comparison between the proposed protocol and the battery-limited OSTS and OCJS protocols [38]. We not only evaluate the confidentiality performance but also pay attention to the transmission performance. To repay the friendly jammer, PUs relax the interference threshold, which leads to reducing the $R_{P}$. Therefore, we set the rate of the PUs of the battery-limited OSTS, OCJS and the proposed model to $R_{P}=0.5\mathrm{Bit}/s/\mathrm{Hz}$. We assume that $R_{S}=0.5\mathrm{Bit}/s/\mathrm{Hz}$, K=3, η=0.7, μ=0.5, T=1s, ξ=0.5, $I=P_{P}$ ($r_{2}=10\log\left(I/N_{0}\right)$), $r_{3}=10\log\left(P_{0}/N_{0}\right)=5dB$ and the channel coefficients $\sigma_{P}^{2}$, $\sigma_{\mathrm{SD}}^{2}$, $\sigma_{\mathrm{PB}}^{2}$, $\sigma_{\mathrm{PE}}^{2}$, $\sigma_{PS_{i}}^{2}$ and $\sigma_{PS_{n}}^{2}$ are normalized to 1, $\sigma_{S_{n}E}^{2}=3$, $\sigma_{S_{i}E}^{2}=1.5$ and $\sigma_{S_{i}}^{2}=4$ in our experiments.

**Figure 3:**
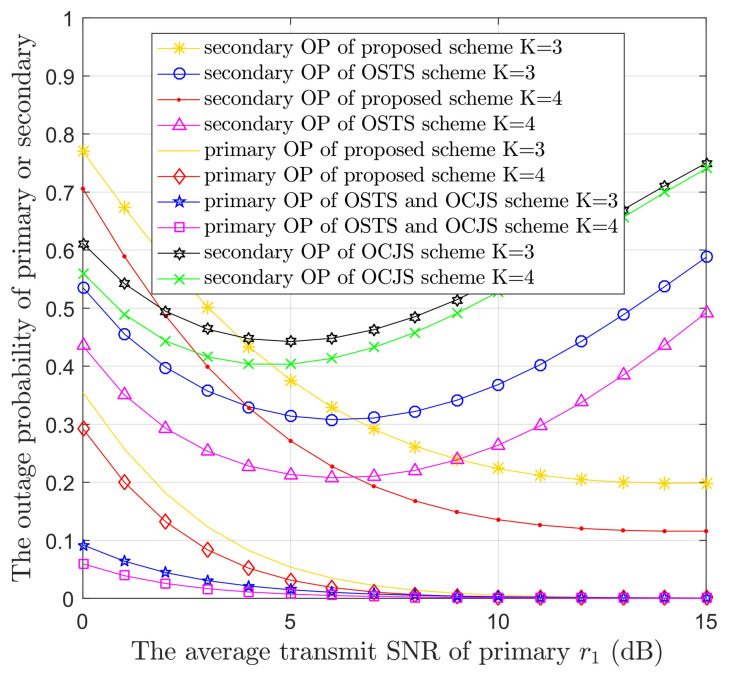
The OPs of PUs or SUs versus the average transmit SNR of primary ($r_{1}$) in the three protocols with different the number of STs (*K*).

Figure 3 displays the OPs of PUs or SUs versus $r_{1}$ ($r_{1}=10\log\left(P_{P}/N_{0}\right)$) of the OSTS and OCJS models as well as the proposed model with different values of *K*. In order to analyze the gain caused by the increase of *K* value, we can set that K=3,4, and all transmission performances are ameliorated with the increase of *K* as a result of the multi-user diversity gain. The primary transmission performance improved with the increase of $r_{1}$ in the three models. This is because primary users can obtain more primary information in the high SNR. However, the OSTS and OCJS schemes can offer a lower outage probability than the proposed scheme. This is because the proposed scheme uses the EH technology of time allocation, which causes the instantaneous capacity of the PS → PD link to become smaller and does not meet the minimum transmission rate of the primary system, resulting in transmission interruption. In addition, with the increase of SNR, the OPs of SUs of three models first decline and then raise. The declining trend is due to the increase of interference threshold $\left(r_{2}=10\log\left(I/N_{0}\right)\right)$ as the SNR of the primary network $\left(r_{1}=10\log\left(P_{P}/N_{0}\right)\right)$ increases, so that the power of the secondary transmitter increases and more information can be transmitted. In addition, the raising trend is because the interference at secondary users also increases when $r_{1}$ is too large. Furthermore, due to the EH technology, the power of STs becomes larger, and the proposed scheme can achieve better secondary transmission performance under the condition of high SNR.

**Figure 4:**
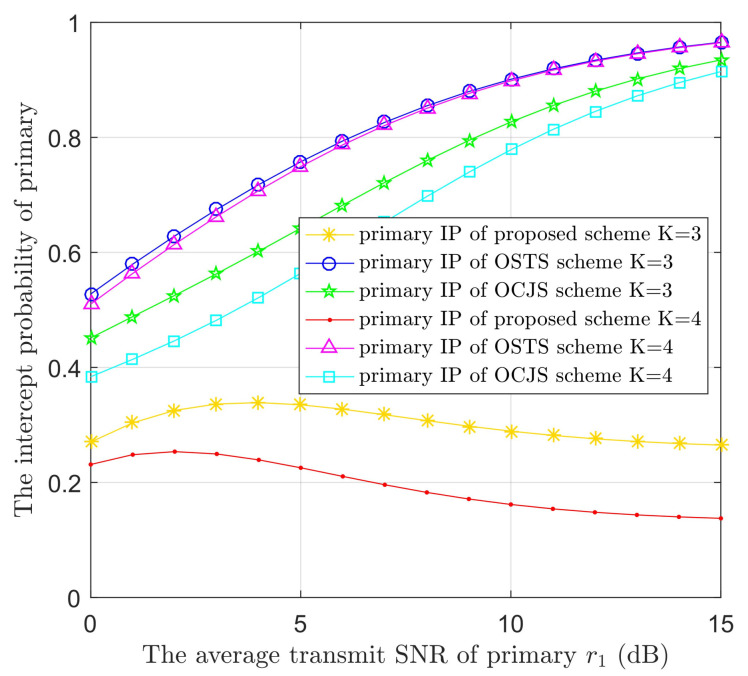
The IPs of the PUs versus the average transmit SNR of primary ($r_{1}$) in the three protocols with different numbers of STs (*K*).

**Figure 5:**
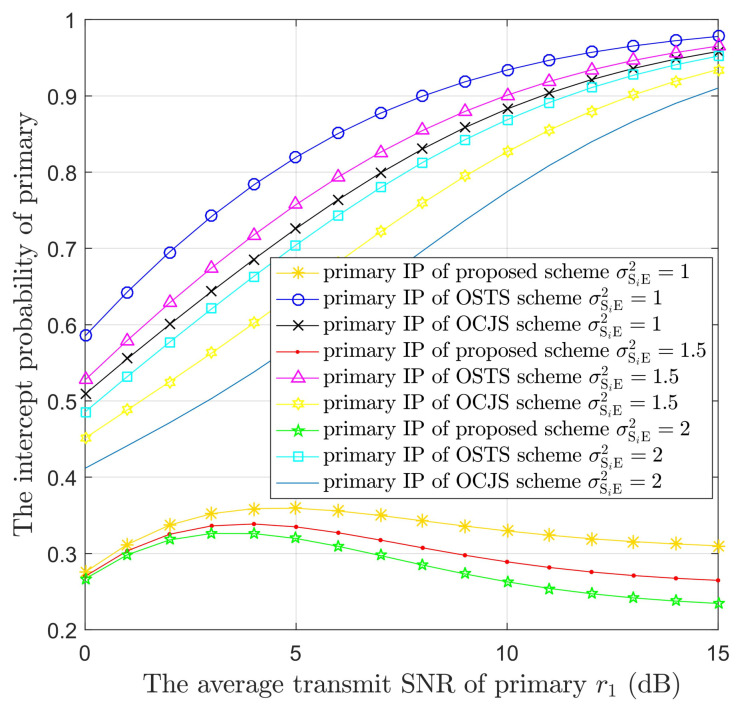
The IPs of the PUs versus the average transmit SNR of primary ($r_{1}$) in the three protocols with different channel coefficient ($\sigma_{S_{i}E}^{2})$) values.

**Figure 6:**
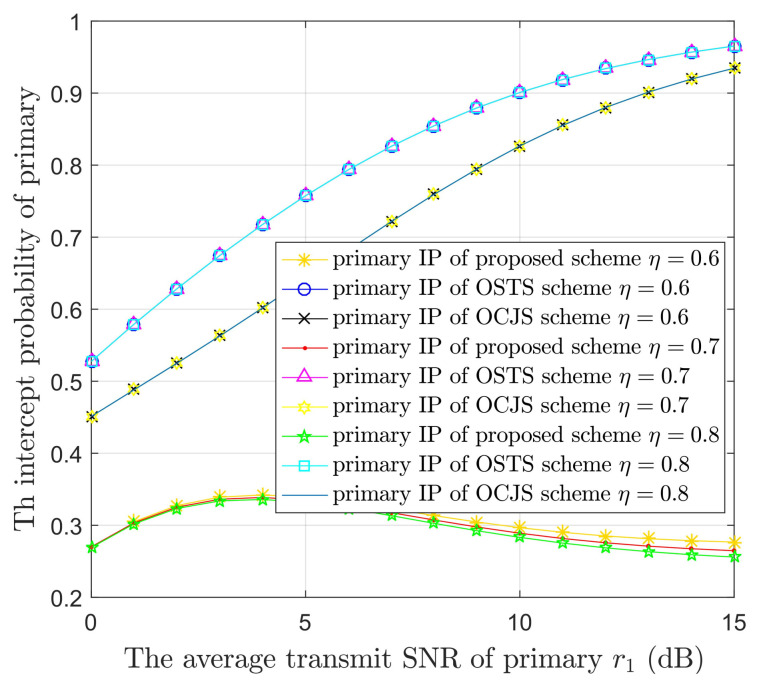
The IPs of the PUs versus the average transmit SNR of primary ($r_{1}$) in the three protocols with different energy transfer efficiency (η) values.

**Figure 7:**
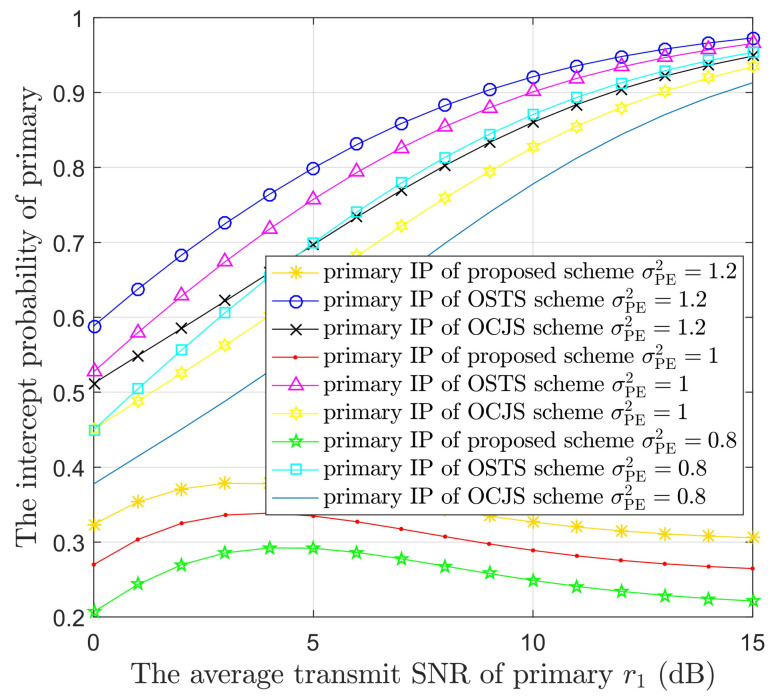
The IPs of the PUs versus the average transmit SNR of primary ($r_{1}$) in the three protocols with different channel coefficient ($\sigma_{\mathrm{PE}}^{2}$) values.

Figure 4 displays the IPs of PUs versus $r_{1}$ in the three models with different values of *K*. The number of STs is 3 or 4. According to Figure 4, the PUs’ confidentiality performance is ameliorated in three models with the increase of the number of STs. The confidentiality performance of the proposed model is better than that of the battery-limited OSTS and OCJS models. In addition, the IPs of PUs decline with the raise of $r_{1}$ in the proposed model. This is because the ST ${}_{i}$ can transmit AN to prevent eavesdropping and the ST ${}_{n}$ has the best channel to E; then, ST ${}_{n}$ can also increase interference with the eavesdropper. The short increasing trend is due to the lower power and poor performance of STs in the small value range of $r_{1}$, and the interference to E is decreased. However, in the battery-limited OSTS and OCJS models, the confidentiality performance of the PUs increases as the $r_{1}$ increases. This is because the eavesdropper can obtain more primary information by a higher value of $r_{1}$ and ${\mathrm{ST}}_{i}$ has only a small part of power to transmit AN. This is the cause of the security performance deteriorating sharply. These phenomena are also shown in Figure 5, Figure 6 and Figure 7.

Figure 5 illustrates the IPs of PUs versus $r_{1}$ in the three models with different $\sigma_{S_{i}E}^{2}$. Since the interference channel gain of $\sigma_{S_{i}E}^{2}$ is greater than the channel gain of $\sigma_{PE}^{2}$ and smaller than the channel gain of $\sigma_{S_{n}E}^{2}$, therefore, the channel coefficient $\sigma_{S_{i}E}^{2}$ is equal to 1, 1.5 or 2. As described in Figure 5, the proposed model is able to offer better confidentiality performance in the same channel coefficient compared to [38]. Moreover, the confidentiality performance is ameliorated obviously in two models as the value of $\sigma_{S_{i}E}^{2}$ becomes larger. This is because the ${\mathrm{ST}}_{i}$→E link has better channel condition in a larger $\sigma_{S_{i}E}^{2}$ value. In other words, the ${\mathrm{ST}}_{i}$ transmits more interference to the eavesdropper as the value of $\sigma_{S_{i}E}^{2}$ increases. In the proposed model, both ${\mathrm{ST}}_{n}$ and ${\mathrm{ST}}_{i}$ interfere with the E. Nevertheless, the interference to E from ${\mathrm{ST}}_{i}$ is worse than that from ${\mathrm{ST}}_{n}$. Hence, the PUs’ secrecy performance is improved slightly. Moreover, the battery-limited OSTS and OCJS models interfere with E only from ${\mathrm{ST}}_{i}$. However, the OCJS method selects the secondary transmitter that can provide the optimal intercept probability to E, so the security performance of OCJS is better than that of OSTS.

Figure 6 illustrates the IPs of PUs versus $r_{1}$ in the three models with different η. The energy transfer efficiency η is equal to 0.6, 0.7 or 0.8, where the specific values set is referred to [30,41]. The confidentiality performance is ameliorated in the proposed model as η is raised. This is because the larger value of η means more energy can be used for the artificial noise transmission. Namely, STs transmit more interference to the eavesdropper as the value of η increases. Nonetheless, the primary security performance remains unchanged in the battery-limited OSTS and OCJS models. This is because energy harvesting is not considered in the OSTS and OCJS models. Then, the change of energy transfer efficiency η has no effect on the intercept probability.

Figure 7 illustrates the IPs of PUs versus $r_{1}$ in the three models with different values of $\sigma_{\mathrm{PE}}^{2}$. The channel coefficient $\sigma_{\mathrm{PE}}^{2}$ equals to 1.2, 1 or 0.8. According to Figure 7, the confidentiality performance deteriorated with the increase of channel coefficient $\sigma_{\mathrm{PE}}^{2}$ in the same model. This is because the PS→E link has better channel conditions in a lager $\sigma_{\mathrm{PE}}^{2}$ value. In other words, the eavesdropper can obtain more primary information via a larger $\sigma_{\mathrm{PE}}^{2}$ value. Furthermore, the proposed model also can provide the better primary confidentiality performance compared to [38].

## 6. Conclusions

The paper investigated the PLS of the underlying model of cognitive Internet of Things. We proposed an ST cooperative jammer selection transmission and energy-harvesting protocol to safeguard PUs and prevent eavesdropping. We also conducted a detailed theoretical study on the performance for the proposed protocol and the battery-limited OSTS protocol. The closed-form expressions of OP and IP of the above two models in Rayleigh fading channels were derived. Moreover, we also considered the OCJS model for further comparison in the experimental part. The final numerical results illustrate that the proposed protocol has better secrecy performance than the battery-limited OSTS and OCJS models due to ST selection transmission and energy harvesting. In addition, the proposed scheme achieves better secondary transmission performance under the condition of high primary SNR. In addition, multi-user diversity technology can be also used to improve system performance. Furthermore, we also analyzed other parameters that influence the system performance to provide a better understanding of the secrecy of the cognitive IoT with EH.

## Figures and Tables

**Table 1:** Summary of some related works.

Methods	Major Domain	Metrics	Technique	Main Contributions
[15]	PLS	perfect secrecy capacity	algebraic Riccati equation	The perfect secrecy capacity of multi antenna MIMO channel is calculated.
[21]	PLS	secrecy outage probability	friendly jammer, AN	Legitimate users achieved better secrecy performance.
[32]	PLS, CR	throughput	CSS	According to the appropriate *K* value, an optimal CSS strategy is developed to maximize throughput.
[33]	PLS, EH, CR	secrecy outage probability (SOP)	relay, jammer	It deduced the exact and asymptotic expressions of SOP.
[34]	PLS, EH, CR	OP, IP	SRT	The results revealed that there is a constraint relationship between reliability and safety.
[35]	PLS, EH, CR	OP, IP	SRT	It proposed two user-scheduling methods to improve the performance of secondary users.
Ours	PLS, EH, CR	OP, IP	dual secondary transmitter selection, AN	It improved the security performance of primary users and the transmission performance of secondary users.

## Data Availability

Not applicable.

## Appendix A

From (27), by using (7) and letting $\Delta_{P}=2^{\frac{R_{P}}{\left(1-\mu \right)T}}-1$, (27) is rewritten as

(A1) PrQ=∅=∏i=1KPrCP2<RP=∏i=1KPrPSihSiD2+N0PPhP2>1ΔP=∏i=1KPrminIhSiD2,ημPPhPSi2+P01−μhSiD2+N0>PPhP2ΔP=∏i=1KPrIhSiD2·hSiD2+N0>PPhP2ΔP︸Ch1=×PrημPPhPSi2+P01−μ·hSiD2+N0>PPhP2ΔP︸Ch2.

Here, ${\left|h_{PS_{i}}\right|}^{2}$, ${\left|h_{P}\right|}^{2}$, and ${\left|h_{S_{i}D}\right|}^{2}$ are independently and exponentially distributed random variables with parameters 11σPSi2σPSi2, 11σP2σP2, and 11σSiD2σSiD2, respectively. Let $X_{1}={\left|h_{PS_{i}}\right|}^{2}$, $X_{2}={\left|h_{P}\right|}^{2}$, and $X_{3}={\left|h_{S_{i}D}\right|}^{2}$, and their PDFs are given by (1). Thus, Ch1 can be derived as

(A2) Ch1=PrI+N0>PPX2PPX2ΔPΔP=1−PrI+N0<PPX2PPX2ΔPΔP=1−∫ΔPI+ΔPN0PP∞1σP2e−x2σP2dx2=1−e−ΔPI+ΔPN0PPσP2.

Meanwhile, Ch2 can be derived as

(A3) $$\begin{array}{cc} \mathrm{Ch}2 & =\mathrm{Pr}\left\{\frac{\eta \mu P_{P}X_{1}X_{3}+P_{0}X_{3}}{1-\mu }+N_{0}>\frac{P_{P}X_{2}}{\Delta_{P}}\right\}\\ \; & =1-\mathrm{Pr}\left\{\frac{\eta \mu P_{P}X_{1}X_{3}+P_{0}X_{3}}{1-\mu }+N_{0}<\frac{P_{P}X_{2}}{\Delta_{P}}\right\}\\ \; & =1-\int_{0}^{\infty }\frac{1}{\sigma_{S_{i}D}^{2}}e^{-\frac{x_{3}}{\sigma_{S_{i}D}^{2}}}dx_{3}\int_{0}^{\infty }\frac{1}{\sigma_{PS_{i}}^{2}}e^{-\frac{x_{1}}{\sigma_{PS_{i}}^{2}}}dx_{1}\int_{\frac{\left(\eta \mu P_{P}x_{3}x_{1}+P_{0}x_{3}\right)\Delta_{P}}{\left(1-\mu \right)P_{P}}+\frac{\Delta_{P}N_{0}}{P_{P}}}^{\infty }\frac{1}{\sigma_{P}^{2}}e^{-\frac{x_{2}}{\sigma_{P}^{2}}}dx_{2}\\ \; & =1-\int_{0}^{\infty }\frac{1}{\sigma_{S_{i}D}^{2}}e^{-\frac{x_{3}}{\sigma_{S_{i}D}^{2}}}dx_{3}\int_{0}^{\infty }\frac{1}{\sigma_{PS_{i}}^{2}}e^{-\frac{x_{1}}{\sigma_{PS_{i}}^{2}}}dx_{1}\times e^{-\left(\frac{\left(\eta \mu P_{P}x_{3}x_{1}+P_{0}x_{3}\right)\Delta_{P}}{\left(1-\mu \right)P_{P}}+\frac{\Delta_{P}N_{0}}{P_{P}}\right)}\\ \; & =1+\frac{\left(1-\mu \right)\sigma_{P}^{2}}{\eta \mu \sigma_{S_{i}D}^{2}\sigma_{PS_{i}}^{2}\Delta_{P}}e^{-\frac{N_{0}\Delta_{P}}{P_{P}\sigma_{P}^{2}}+\frac{\left(1-\mu \right)P_{P}\sigma_{P}^{2}+\Delta_{P}P_{0}\sigma_{S_{i}D}^{2}}{\eta \mu \sigma_{S_{i}D}^{2}\sigma_{PS_{i}}^{2}\Delta_{P}P_{P}}}\times \mathrm{Ei}\left(-\frac{\left(1-\mu \right)P_{P}\sigma_{P}^{2}+\Delta_{P}P_{0}\sigma_{S_{i}D}^{2}}{\eta \mu \sigma_{S_{i}D}^{2}\sigma_{PS_{i}}^{2}\Delta_{P}P_{P}}\right), \end{array}$$

where $\mathrm{Ei}\left(x\right)=\int_{-\infty }^{x}\frac{1}{u}e^{u}du=\gamma +\ln\left(-x\right)+\sum_{n=1}^{\infty }\frac{x^{n}}{n\;\cdot \;n!},x<0$, γ is the Euler’s constant.

From (30), by using (7) and letting $\Delta_{S}=2^{\frac{R_{S}}{\left(1-\mu \right)T}}-1$ and $a_{1}=\frac{{\left|h_{S_{i}}\right|}^{2}}{P_{P}{\left|h_{\mathrm{PB}}\right|}^{2}+N_{0}}$, (32) can be rewritten as

(A4) PrΨS|Q=Ql=∏i=1L1−PrminIhSiD2,ημPPhPSi2+P01−μa1>ΔS=∏i=1L1−PrhSi2·IIhSiD2hSiD2PPhPB2+N0>ΔS︸Ch3=×PrhSi2ημPPhPSi2+P0ημPPhPSi2+P01−μ1−μPPhPB2+N0>ΔS︸Ch4.

Here, ${\left|h_{\mathrm{PB}}\right|}^{2}$ and ${\left|h_{S_{i}}\right|}^{2}$ are independently and exponentially distributed random variables with parameters 11σPB2σPB2 and 11σSi2σSi2, respectively. Let $X_{4}={\left|h_{\mathrm{PB}}\right|}^{2}$ and $X_{5}={\left|h_{S_{i}}\right|}^{2}$; their PDFs are given by (1). Let Y1=X5Y1=X5X3X3; the PDF of variable $Y_{1}$ is derived in Appendix B from (A16). By using $f_{Y_{1}}\left(y_{1}\right)$, Ch3 can be derived as

(A5) $$\begin{array}{cc} \mathrm{Ch}3 & =1-\mathrm{Pr}\left\{I\;\cdot \;W_{1}<\Delta_{S}P_{P}X_{4}+\Delta_{S}N_{0}\right\}\\ \; & =1-\int_{0}^{\infty }f_{Y_{1}}\left(y_{1}\right)dy_{1}\int_{\frac{Iy_{1}-\Delta_{S}N_{0}}{\Delta_{S}P_{P}}}^{\infty }\frac{1}{\sigma_{\mathrm{PB}}^{2}}e^{-\frac{x_{4}}{\sigma_{\mathrm{PB}}^{2}}}dx_{4}\\ \; & =1-e^{\frac{N_{0}}{P_{P}\sigma_{\mathrm{PB}}^{2}}}\int_{0}^{\infty }\frac{\sigma_{S_{i}}^{2}\sigma_{S_{i}D}^{2}}{{\left(\sigma_{S_{i}}^{2}+\sigma_{S_{i}D}^{2}y_{1}\right)}^{2}}e^{-\frac{I}{\Delta_{S}P_{P}\sigma_{\mathrm{PB}}^{2}}y_{1}}dy_{1}\\ \; & =1-\frac{\sigma_{S_{i}}^{2}}{\sigma_{S_{i}D}^{2}}e^{\frac{N_{0}}{P_{P}\sigma_{\mathrm{PB}}^{2}}}\left[\frac{I}{\Delta_{S}P_{P}\sigma_{\mathrm{PB}}^{2}}\right.\left.e^{\frac{I\sigma_{S_{i}}^{2}}{\Delta_{S}P_{P}\sigma_{\mathrm{PB}}^{2}\sigma_{S_{i}D}^{2}}}\mathrm{Ei}\left(\frac{-I\sigma_{S_{i}}^{2}}{\Delta_{S}P_{P}\sigma_{\mathrm{PB}}^{2}\sigma_{S_{i}D}^{2}}\right)+\frac{\sigma_{S_{i}D}^{2}}{\sigma_{S_{i}}^{2}}\right]. \end{array}$$

In addition, Ch4 can be derived as

(A6) Ch4=1−PrημPPX5X1+P0X51−μ<ΔSPPX4+ΔSN0=1−∫0∞1σSi2e−x5σSi2dx5∫0∞1σPSi2e−x1σPSi2dx1∫ημPPx5x1+P0x51−μΔSPP−N0PP∞1σPB2e−x4σPB2dx4=1−1σSi2eN0PPσPB2∫0∞1−μΔSσPB21−μΔSσPB2+ημσPSi2x5e−x5σSi2−P0x51−μΔSPPσPB2dx5=1+1−μΔSσPB2ημσSi2σPSi2e1σSi2+P01−μΔSPPσPB21−μΔSσPB2ημσPSi2+N0PPσPB2=×Ei−1σSi2+P01−μΔSPPσPB21−μΔSσPB2ημσPSi2.

From (36), let $X_{6}={\left|h_{\mathrm{PE}}\right|}^{2}$, $X_{7}={\left|h_{S_{i}E}\right|}^{2}$, $X_{8}={\left|h_{S_{n}E}\right|}^{2}$, $X_{9}={\left|h_{PS_{n}}\right|}^{2}$, and the PDF of variable $Y_{2}$ is derived in Appendix B from (A16) to (A17). By using $f_{Y_{2}}\left(y_{2}\right)$, Ch5 and Ch6 can be derived as (A7) and (A8), respectively.

(A7) Ch5=PrIY2+ημPPX9X81−μ+N0>PPX6PPX6ΔPΔP=1−∫0∞σSiE2σSiD2σSiE2+σSiD2y22dy2∫0∞1σPSn2e−x9σPSn2dx9∫0∞1σSnE2e−x8σSnE2dx8=×∫ΔPIy2PP+ημΔPx8x91−μ+N0ΔPPP∞1σPE2e−x6σPE2dx6=1−∫0∞σSiE2σSiD2σSiE2+σSiD2y22dy2∫0∞1σPSn2e−x9σPSn2dx9∫0∞1σSnE2e−x8σSnE2dx8=×e−ΔPIy2PP+ημΔPx8x91−μ+N0ΔPPP=1+e−N0ΔPPPσPE2+1−μσPE2ημΔPσSnE2σPSn21−μσPE2ημΔPσSnE2σPSn2Ei−1−μσPE2ημΔPσSnE2σPSn2=×σSiE2σSiD2IΔPPPσPE2eIΔPσSiE2PPσPE2σSiD2Ei−IΔPσSiE2PPσPE2σSiD2+σSiD2σSiE2,

(A8) Ch6=PrημPPX1+P0X7+ημPPX9X81−μ+N0>PPX6ΔP=1−∫0∞1σSnE2e−x8σSnE2∫0∞1σPSn2e−x9σPSn2dx9dx8∫0∞1σPSi2e−x1σPSi2∫0∞1σSiE2e−x7σSiE2dx7dx1=×∫P0ΔPx71−μPP+ημΔPx1x71−μ+ημΔPx9x81−μ+ΔPN0PP∞1σPE2e−x6σPE2dx6=1−∫0∞1σSnE2e−x8σSnE2∫0∞1σPSn2e−x9σPSn2dx9dx8∫0∞1σPSi2e−x1σPSi2∫0∞1σSiE2e−x7σSiE2dx7dx1=×e−P0ΔPx71−μPP+ημΔPx1x71−μ+ημΔPx9x81−μ+ΔPN0PP=1−1−μσPE2ημΔP21σPSn2σPSi2σSiE2σSnE2e−N0ΔPPPσPE2+P0ΔPσSiE2+1−μPPσPE2ημΔPPPσSiE2σPSi2+1−μσPE2ημΔPσPSn2σSnE2=×Ei−P0ΔPσSiE2+1−μPPσPE2ημΔPPPσSiE2σPSi2Ei−1−μσPE2ημΔPσPSn2σSnE2.

By using (16), (41) can be rewritten as,

(A9) PrQOSTS=∅=∏i=1KPrCP2OSTS<RP=∏i=1KPrξPSiOSTShSiD2+N0PPhP2>1ρP=∏i=1KPrminIhSiD2,P0·ξhSiD2+N0>PPhP2ρP=∏i=1KPrξIhSiD2·hSiD2+N0>PPhP2ρP︸Ch7=×PrξP0·hSiD2+N0>PPhP2ρP︸Ch8.

Hence, Ch7 is derived as

(A10) Ch7=PrξI+N0>PPX2PPX2ρPρP=1−PrξI+N0<PPX2PPX2ρPρP=1−∫ρPξI+ρPN0PP∞1σP2e−x2σP2dx2=1−e−ρPξI+ρPN0PPσP2.

Ch8 can be derived as follows:

(A11) Ch8=PrξP0X3+N0>PPX2>PPX2ρPρP=1−PrξP0X3+N0<PPX2<PPX2ρPρP=1−∫0∞1σSiD2e−x3σSiD2dx3∫ξP0x3ρPPP+ρPN0PP∞1σP2e−x2σP2dx2=1−∫0∞1σSiD2e−x3σSiD2dx3×e−ξP0x3ρPPP+ρPN0PP=1+PPσP2PPσP2+ρPξP0σSiD2e−N0ρPPPσP2.

From (44), we can obtain

(A12) Ch9=1−PrξIY1ξIY1PPρSPPρS−N0N0PPPP<X4=1−∫0∞σSi2σSiD2σSi2+σSiD2y12dy1∫ξIy1PPρS−N0PP∞1σPB2e−x4σPB2dx4=1−∫0∞σSi2σSiD2σSi2+σSiD2y12dy1×e−ξIy1PPρS−N0PP=1−σSi2σSiD2eN0PPσPB2ξIρSPPσPB2eξIσSi2ρSPPσPB2σSiD2×Ei−ξIσSi2ρSPPσPB2σSiD2+σSiD2σSi2,

(A13) Ch10=PrX5>PPρSX4+ρSN0ξP0=ξP0σSi2e−ρSN0ρSN0P0σSi2P0σSi2ξP0σSi2+PPρSσPB2.

Considering Y2=hSiE2hSiE2hSiD2hSiD2, then using the PDF of $Y_{2}$, which is calculated in Appendix B, from (46), we can obtain

(A14) Ch11=PrPPhPE2<ρPIhSiE2IhSiE2hSiD2hSiD2+N0=∫0∞σSiE2σSiD2σSiE2+σSiD2y22dy2∫0IρPy2PP+ρPN0PP1σPE2e−x6σPE2dx6=1−σSiE2σSiD2e−ρPN0PPσPE2IρPPPσPE2eIρPσSiE2PPσPE2σSiD2×Ei−IρPσSiE2PPσPE2σSiD2+σSiD2σSiE2,

(A15) Ch12=PrPPhPE2P0hSiE2+N0<ρP=1−PPσPE2e−ρPN0ρPN0PPσPE2PPσPE2PPσPE2+P0ρPσSiE2.

## Appendix B

According to $X_{5}={\left|h_{S_{i}}\right|}^{2}$, $X_{3}={\left|h_{S_{i}D}\right|}^{2}$, and (1), let $Y_{1}=X_{5}/X_{3}$; then, the PDF of $Y_{1}$ can be derived as

(A16) FY1y1=PrX5X3<y1=∫0∞1σSiD2e−x3σSiD2∫0x4y11σSi2e−x5σSi2dx5dx3=1−σSi2σSi2σSi2+σSiD2y1σSi2+σSiD2y1⇒fY1y1=σSiD2σSi2σSiD2σSi2σSi2+σSiD2y12σSi2+σSiD2y12.

Let Y2=X7Y2=X7X3X3, where $X_{7}={\left|h_{S_{i}E}\right|}^{2}$. Similar to the derivation of the PDF of $Y_{1}$, the PDF of $Y_{2}$ can obtained by (A17).

(A17) fY2y2=σSiD2σSiE2σSiD2σSiE2σSiE2+σSiD2y22σSiE2+σSiD2y22.
